# Cyanobacterial Cultures, Cell Extracts, and Individual Toxins Decrease Photosynthesis in the Terrestrial Plants *Lactuca sativa* and *Zea mays*

**DOI:** 10.3390/plants13223190

**Published:** 2024-11-13

**Authors:** Scott A. Heckathorn, Clare T. Muller, Michael D. Thomas, Emily P. Vining, Samantha Bigioni, Clair Elsie, J. Thomas Franklin, Emily R. New, Jennifer K. Boldt

**Affiliations:** 1 Department of Environmental Sciences, University of Toledo, Toledo, OH 43606, USA; clare.muller@utoledo.edu (C.T.M.); michael.thomas11@utoledo.edu (M.D.T.); emily.vining@utoledo.edu (E.P.V.); emily.new@rockets.utoledo.edu (E.R.N.); 2 Ottawa Hills High School, Ottawa Hills, OH 43606, USA; sbigioni@gmail.com (S.B.); franklin.john.thomas@gmail.com (J.T.F.); 3 Sylvania High School, Sylvania, OH 43560, USA; caelsie27@gmail.com; 4 Agricultural Research Service, United States Department of Agriculture, Toledo, OH 43606, USA; jennifer.boldt@usda.gov

**Keywords:** anatoxin, beta-methyl-amino-L-alanine, harmful algal blooms, lipopolysaccharide, microcystin, photosynthetic rate

## Abstract

Cyanobacterial harmful algal blooms (cHABs) are increasing due to eutrophication and climate change, as is irrigation of crops with freshwater contaminated with cHAB toxins. A few studies, mostly in aquatic protists and plants, have investigated the effects of cHAB toxins or cell extracts on various aspects of photosynthesis, with variable effects reported (negative to neutral to positive). We examined the effects of cyanobacterial live cultures and cell extracts (*Microcystis aeruginosa* or *Anabaena flos-aquae*) and individual cHAB toxins (anatoxin-a, ANA; beta-methyl-amino-L-alanine, BMAA; lipopolysaccharide, LPS; microcystin-LR, MC-LR) on photosynthesis in intact plants and leaf pieces in corn (*Zea mays*) and lettuce (*Lactuca sativa*). In intact plants grown in soil or hydroponically, overall net photosynthesis (P_n_), but not Photosystem-II (PSII) electron-transport yield (Φ_PSII_), decreased when roots were exposed to cyanobacterial culture (whether with intact cells, cells removed, or cells lysed and removed) or individual toxins in solution (especially ANA, which also decreased rubisco activity); cyanobacterial culture also decreased leaf chlorophyll concentration. In contrast, Φ_PSII_ decreased in leaf tissue vacuum-infiltrated with cyanobacterial culture or the individual toxins, LPS and MC-LR, though only in illuminated (vs. dark-adapted) leaves, and none of the toxins caused significant decreases in in vitro photosynthesis in thylakoids. Principal component analysis indicated unique overall effects of cyanobacterial culture and each toxin on photosynthesis. Hence, while cHAB toxins consistently impacted plant photosynthesis at ecologically relevant concentrations, the effects varied depending on the toxins and the mode of exposure.

## 1. Introduction

Climate change and eutrophication are increasing the frequency and severity of toxin-producing cyanobacterial harmful algal blooms (cHABs) in freshwater systems [1,2]. As a result, many surface and ground-water sources may contain, or even accumulate, cyanobacterial toxins [3,4,5]. Chronic and acute exposure to these toxins pose a significant health risk to humans and other animals, as they may lead to illness or death [6,7,8]. Contaminated food sources may present a significant and growing source of toxin exposure [4,9]. Agricultural plants can accumulate cyanobacterial toxins after irrigation with contaminated water or fertilization with cyanobacteria-based bio-fertilizers, resulting in crop contamination that can exceed safety thresholds for daily tolerable intake (e.g., the World Health Organization’s adult daily tolerable intake for microcystin-LR of 0.04 µg/kg bodyweight); cyanobacterial toxins can also decrease crop productivity [9,10,11,12,13,14].

Cyanobacteria that cause freshwater HABs, such as *Microcystis* and *Anabaena* (also known as *Dolichospermum*) species, often produce an array of toxins, including microcystins (MCs), anatoxin-a (ANA), β-methyl-amino-L-alanine (BMAA), and lipopolysaccharides (LPSs) [4]. These four toxins, which are among the most common in freshwater systems, are known to have different modes of action and toxicity in animals and plants [4,9,15,16,17]. Because cHAB water or cyanobacterial extracts contain multiple toxins, the effects of cHAB-contaminated water or cyanobacterial extracts on plants will likely be complex and more severe compared to the effects of individual toxins (e.g., [18]). Most studies to date examining the effects of cyanobacterial toxins on plants have either treated plants with cyanobacterial extracts or individual toxins, so our understanding of the comparative effects of different toxins or cyanobacterial extracts vs. individual toxins is lacking [4,9,16].

The most common and well-studied cyanobacterial toxins are the microcystins, especially MC-LR. These cyclic heptapeptides are composed of five non-protein (or non-canonical) amino acids and 3-amino-9-methoxy-2,6,8-trimethyl-10-phenyl-4,6-decadienoic acid (ADDA), with hundreds of congeners, resulting from substitutions in the second and fourth positions of the peptide, which vary in toxicity [16,19]. MCs inhibit eukaryotic protein phosphatases 1 (PP1) and 2A (PP2A) [20], which regulate a wide array of cell functions, including in plants [21]. Hence, MCs have wide-ranging negative effects on cells, including causing hyper-phosphorylation of cellular proteins and the generation of reactive oxygen species (ROS), DNA damage, and cell death [4,13]. Many studies have investigated the effects of microcystins on plant growth and function, although few have examined the effects on photosynthesis, with the effects of microcystins ranging from positive to neutral to negative depending on the species, the growth stage, the MC congener, the quantity of the toxin, etc. (reviewed in [4,9,13,16]).

Anatoxin-a (ANA) is a neurotoxic bicyclic amine alkaloid that acts by binding to nicotinic and muscarinic acetylcholine receptors in animals, where it mimics the neurotransmitter acetylcholine [22]. Non-neuronal acetylcholine is present in plant cells, where it may interact with many cellular processes [23]. In plants, nicotinic and muscarinic receptor antagonists, in general, have been shown to reduce cell size, alter cell structure, and affect water uptake [24]. The effects of ANA specifically on plants have rarely been investigated [4,22], but it is known to negatively impact growth and function (e.g., [25,26,27]).

The non-canonical amino acid, BMAA, can interfere with mammalian glutamate receptors [15,17]. BMAA may also be incorporated into proteins during protein synthesis, causing proteins to function improperly; it can induce oxidative stress, and it can likely disrupt N metabolism by interfering with ammonium assimilation [15,17]. BMAA has been shown to bioaccumulate in plants, either in free or protein-associated forms [17,28,29,30]. The effects of BMAA on plant function have rarely been examined [4]. It is likely that BMAA interferes with glutamate receptors in plant cells, but it is difficult to predict how this might affect them, as there are many types and roles of glutamate receptors in plants [17]. One study suggests that BMAA may increase oxidative stress in the aquatic plant, *Ceratophyllum demersum*, via inhibition of oxidative stress enzymes, leading to ROS buildup [31], and another study found that BMAA decreased the growth of *Medicago sativa* seedings at extreme concentrations [32]. On the other hand, it has been hypothesized (but not proven) that BMAA may play a role in the protection of photosynthesis during stress in cyanobacteria [33], but this has not been examined in plants.

Lipopolysaccharides (LPSs) are bacterial endotoxins that comprise a lipid A moiety, a core oligosaccharide, and an O-antigen, and they are found in the cell wall of Gram-negative bacteria, including cyanobacteria. Although the LPS lipid and oligosaccharide components are highly conserved, the O-antigen varies among species, which can affect toxicity. Interestingly, Bernardova et al. found that LPSs from cyanobacteria are similar in toxicity to LPSs from heterotrophic bacteria and that LPSs from cHABs can be more toxic due to the presence of multiple species [34]. In plants, LPSs are detected as a microbe-associated molecular pattern by cells and triggers the release of ROS, elevated intracellular Ca^2+^, and increased expression of kinase-mediated pathways that strengthen defense and resistance to microbial pathogens (e.g., [35,36,37]). Plant perceptions of and reactions to LPSs vary among plant groups and depend on what LPS species is detected [38,39]. Numerous downstream effects of LPSs on of kinase signaling could impact plant function, including photosynthesis, but data on the effects of LPSs on photosynthesis are rare.

Only a small number of studies have examined the effects of cyanobacterial extracts or individual toxins on photosynthesis in general and in plants in particular [4,9,16]. Past studies have examined cHAB toxin effects (mostly microcystins or whole-cell extracts) on various aspects of photosynthesis in organisms ranging from cyanobacteria (e.g., [40]) to photosynthetic protists (e.g., [18,41,42]), aquatic plants (e.g., [25,26,27,43]), and terrestrial plants (e.g., [44,45,46,47]). Most past studies have examined toxin effects only on chlorophyll concentration or Photosystem-II (PSII) function, which do not necessarily reflect the effects on net photosynthesis, and only a few studies have measured the overall net photosynthesis rate (as net CO_2_ uptake or O_2_ evolution) [25,43,44,45,47]. Excluding Chen et al. [43], who examined the effects of MC-LR on rubisco activity, we are aware of no studies that have examined the effects of cHAB toxin effects on CO_2_-fixation reactions in photosynthesis. The effects of cHAB toxins on photosynthesis are often dose- or time-dependent (e.g., [26,27,43]), and they can vary among species within a study (e.g., [41]), the parameters measured (e.g., [chl] vs. PSII [40]), and whole-cell extracts vs. pure toxins (e.g., [18,41]). In some past studies, the concentrations of toxins used were so extremely low or high that they rendered the results of limited usefulness, e.g., positive effects on photosynthesis at up to 1 ng/L MC [41] or negative effects of MC at 2220–100,000 μg/L [27,44,46]. In plants, cHAB toxin effects on photosynthesis are often attributed to indirect damage from ROS (e.g., [26,36,45]) or manifest after several days (e.g., [27,46]), so it is not clear if the cHAB toxins can affect plant photosynthesis directly.

To gain a clearer understanding of the impact of cHAB toxins on plant photosynthesis, we conducted multiple experiments to determine if four common cHAB toxins affect various aspects of photosynthesis (including net photosynthesis, stomatal conductance, PSII of the light reactions, rubisco activity of the CO_2_-fixation reactions, and chlorophyll concentration). Two different species (corn, lettuce) were treated with live cultures, cell extracts, and/or individual pure toxins at ecologically or physiologically relevant concentrations (ANA, BMAA, LPS, MC-LR) either via uptake by the roots in soil or hydroponically grown plants or via direct application to the leaves. To our knowledge, this is the first study to examine the effects of multiple cHAB toxins and cyanobacterial cells or extracts on both light and CO_2_-fixation reactions in plants exposed to toxins through the roots vs. the leaves.

## 2. Materials and Methods

### 2.1. Plant Growth

Corn (*Zea mays* L. cv ‘Bicolor Sh2’), lettuce (*Lactuca sativa* L. cv ‘Casey’), and tomato (*Solanum lycopersicum* L. cv ‘Early Girl’) (Johnny’s Select Seeds, Winslow, ME, USA) were grown from seed in a greenhouse. Greenhouse temperatures ranged from ca. 20–22 °C (night-time low) to ca. 28–32 °C (day-time high) for lettuce and tomato grown in late fall to early spring and from ca. 22–26 °C (night-time low) to ca. 32–38 °C (day-time high) for corn grown in mid spring to late summer. The greenhouse photoperiod was set to 15 h using high-output lamps providing ca. 125 μmol m^−2^ s^−1^ PAR (photosynthetically active radiation) to extend the photoperiod when needed. Otherwise, most greenhouse light was provided by ambient solar radiation, up to ca. 1600 μmol m^−2^ s^−1^ PAR at midday.

Corn was grown in topsoil in drainable plastic pots (10.5-cm wide × 10.5-cm wide × 13.5-cm tall). Lettuce and tomato were grown in 5 cm deep drainable plastic flats filled with calcite clay and peat (1:3, *v*:*v*). Corn plants were watered as needed and fertilized twice weekly with complete nutrient solution [5 mM KNO_3_, 0.5 mM NH_4_Cl, 2 mM KH_2_PO_4_, 2 mM CaCl_2_, 1 mM MgCl, 1 mM K_2_SO_4_, 50 µM Fe (as FeSO_4_ + FeCl_3_, or as Fe-EDTA), 50 µM H_3_BO_3_, 10 µM MnCl_2_, 2.0 µM CuSO_4_, 2.0 µM ZnSO_4_, 0.1 µM NaMoO_4_, and 1 mM MES buffer (pH 6.2 ± 0.2) in RO water]. Lettuce and tomato plants were watered and fertilized similarly [as above but with 4.5 mM KNO_3_, 0.5 mM NH_4_NO_3_, 1 mM urea, 1 mM MgSO_4_]. Corn was used in experiments after it reached the V2 or V3 stages (2 or 3 expanded green leaves), lettuce when it reached ca. 6–10 g fresh mass, and tomato when it had 3 to 5 expanded adult leaves.

For hydroponic experiments, plants were grown as above until the experiment, at which time their roots were rinsed with tap water and they were transferred to glass jars containing nutrient solution. Jars had lids with small holes though which plants were suspended with roots submerged in nutrient solutions, and jars were wrapped in opaque tape to prevent algal growth on roots during the experiment. Hydroponic experiments were conducted in the lab (ca. 22 °C and 15–25% RH) in chemical fume-hoods to prevent human exposure to toxins, and hydroponic solutions were not aerated to prevent airborne release of toxins. Plants were illuminated in the fume-hoods with a high-intensity LED grow lamp (model Pheno 440, Phantom LED, Shoemakersville, PA, USA) providing 700–800 μmol m^−2^ s^−1^ PAR and set to a 14 h photoperiod. Plants were given 7–10 days to acclimate to the lab prior to starting the experiments. During multi-day experiments, plants were rotated ca. daily to avoid position effects. Because corn did not grow well in unaerated hydroponics, we used lettuce in such experiments lasting more than 24 h.

### 2.2. Cyanobacterial Growth

*Microcystis aeruginosa* (strains LB 2385 + LB 3037, UTEX Culture Collection, utex.org, Austin, TX, USA) was either grown in the greenhouse (experiments 1 and 2, Table 1) or in the lab (experiment 4). *Anabaena flos-aquae* (strain 2557, UTEX, or 15170, Carolina Biological, Burlington, NC, USA) was grown in the lab. “Greenhouse” *M. aeruginosa* was grown in 20 L white polypropylene carboys (ca. 40% PAR transmittance), while lab-grown cultures of both species were grown in glass flasks in a sterile hood at 22–23 °C with natural light provided by a nearby window plus light from a fluorescent bulb set to a 14 h photoperiod (yielding up to 35–50 μmol m^−2^ s^−1^ PAR). Except for experiment 3 below, cultures were grown in nutrient solution containing 1 mM KNO_3_, 0.1 mM NH_4_Cl, 0.1 mM KH_2_PO_4_, 250 μM CaCl_2_, 125 μM MgCl, 125 μM K_2_SO_4_, 10 µM FeSO_4_, 50 µM H_3_BO_3_, 10 µM MnCl_2_, 2.0 µM CuSO_4_, 2.0 µM ZnSO_4_, 0.1 µM NaMoO_4_, and 1 mM HEPES buffer (pH 7.3 ± 0.2) in RO water. For experiment 3 below, cyanobacteria were grown in river water (Ottawa River, University of Toledo, Toledo, OH, USA), filtered as below, to which was added 0.08 g commercial plant food (Miracle-Gro water-soluble plant food for vegetables and herbs; Marysville, OH, USA; yielding pH 7.75 ± 0.1 and 1 mM N). Cyanobacterial cultures were continuously aerated using aquarium pumps and tubing. Cyanobacterial cultures were used in experiments when the chlorophyll (chl) concentration reached 0.04–0.10 absorbance units measured at 665 nm with a spectrophotometer (=0.56–1.4 μg/mL chl), which would represent a severe cyanobacterial bloom in coastal water [48]. To obtain cell-free culture, cells were removed by filtering through a 0.45 μm filter (model GF/F, Whatman, China). To obtain a lysed-cell and cell-free culture (containing whole-cell extracts), cells were first removed from cyanobacterial culture, and then the filters with collected cells were frozen at −70 °C. Filters were then shaken in a closed glass jar until the filters disintegrated either in deionized water or in a small volume of ethanol (25 mL per 1500 mL starting culture) followed by deionized water (100 mL) plus Tween-20 non-ionic detergent (3 drops per 1500 mL starting culture). The lysed-cell extract was then filtered as above, and this filtrate was added to the original cell-free filtrate. For the soil experiments, cyanobacterial cultures (whole-cell, cells removed, and cells lysed and removed) were adjusted to match the control nutrient solution for pH; for hydroponics and leaf-disc experiments, lysed and cell-free extract was adjusted to match the control nutrient solution for pH, NO_3_, and NH_4_.

### 2.3. Soil-Grown Corn Treated with Cyanobacterial Culture for 10–14 d

For corn plants in soil (V2 or V3 stage), experiments were conducted in the greenhouse, during which roots were watered every three days (starting with day 0) with 300 mL cyanobacterial nutrient solution (controls) or cyanobacterial culture applied to the soil (experiment 1: *M. aeruginosa,* whole-cell culture; experiment 2: *M. aeruginosa,* whole-cell vs. cells removed; experiment 3: *A. flos-aquae* cells lysed and removed; Table 1). At the end of experiments 1 and 3, plants were harvested, separated into roots and shoots, and weighed to obtain fresh and/or dry mass (the latter after drying for 48 h at 70 °C).

### 2.4. Corn and Lettuce Leaves Treated with Cyanobacterial Culture or Toxins for 1–3 h

Recently expanded leaves of lettuce and corn were used for the experiments. Lettuce leaves were sampled using a circular cutter (3 cm diameter) to create uniform discs, while rectangular sections (ca. 1 × 3 cm) cut with scissors from the middle of corn leaves were used. Leaf pieces were submerged in treatment solutions containing 0.01% Tween detergent, and then leaf tissue was vacuum-infiltrated (at no more than 30 cm Hg) in a chamber for ca. 10 min until leaves were fully infiltrated (determined by color change of leaves). Infiltrated discs were then placed on top of a thin layer of treatment solution in clear plastic Petri dishes with lids, either uncovered (light-adapted) or covered with aluminum foil (dark-adapted). Because lettuce was more amenable to vacuum-infiltration, we used lettuce in most of the infiltration experiments.

For corn and lettuce treated with cyanobacterial culture (experiment 5), leaf tissue was infiltrated with cyanobacterial nutrient solution (controls) or *M. aeruginosa* or *A. flos-aquae* culture (cells lysed and removed; two separate batches of each species for lettuce). Light-adapted leaf discs were incubated for 1 or 3 h, either under 750 ± 25 µmol m^−2^ s^−1^ PAR or in the dark, after which Photosystem-II (PSII) function was measured (see below).

For lettuce treated with individual pure toxins (experiment 7), leaf tissue from greenhouse-grown plants was infiltrated with deionized water (controls) or toxins in deionized water (1 μM ANA, BMAA, MC or 50 µg mL^−1^ LPS) (experiment 7) [ANA/BMAA/MC-LR (products 14609/2387/10007188) from Cayman Chemical, Ann Arbor, MI, USA; LPS from *E. coli* (product L2630) from Sigma-Aldrich, St. Louis, MO, USA]. These ANA, BMAA, and MC-LR concentrations are towards the high end of levels found during naturally occurring HABs [4,22,33], while the LPS concentration was based on previous studies documenting plant sensitivity to the toxin [36,38,39]. Leaves were incubated under 450 µmol m^−2^ s^−1^ PAR or in the dark for 2 h. After incubation, PSII function was measured as below. To further understand the effects of toxin concentration and light dependency on PSII in lettuce leaf discs, an additional experiment was conducted in a similar fashion to the one described above. In this second experiment, leaf discs were infiltrated with either deionized water (controls), 1 µM MC-LR, 10 µM MC-LR, 50 µg mL^−1^ LPS, or 250 µg mL^−1^ LPS. Light-adapted leaf discs were incubated for 3 h, either under 750 ± 25 µmol m^−2^ s^−1^ PAR or in the dark, after which PSII function was measured.

### 2.5. Hydroponic Plants Treated with Cyanobacterial Culture or Individual Toxins

For corn in hydroponics, plants were grown as above to the V2 stage and then transplanted into 500 mL glass jars filled with 475 mL unaerated nutrient solution for seven days to acclimate (experiment 4). Just before initiating the treatments, the plants were transferred to fresh solutions containing either nutrient solution without toxin (controls) or lysed-cell cell-free culture of *M. aeruginosa* or *A. flos-aquae*. After 24 h of treatment, photosynthetic measurements were made on leaves of intact plants (as below).

For lettuce in hydroponics, plants were grown as above to the 4-leaf stage and then transplanted into 250 mL glass jars filled with 225 mL unaerated nutrient solution and allowed seven days to acclimate (experiment 6). Just before initiating the treatments, the initial fresh weights of the plants were obtained. Plants were then transferred to fresh solutions containing either nutrient solution without toxin (controls) or with ANA (0.5 µM), BMAA (0.5 µM), LPS (25 µg mL^−1^), or MC-LR (0.5 µM). Solution levels in jars were maintained daily by refilling with deionized water. After seven days, photosynthetic measurements were made on leaves of intact plants (as below), and then plants were harvested to obtain the final fresh mass and calculate plant growth during the experiment (final mass minus initial mass = ΔFW). Because this experiment was conducted in a chemical fume-hood for safety, this limited the number of replicates per treatment, thereby limiting the statistical power; consequently, this entire experiment (minus the cyanobacterial extract treatment) was repeated, as discussed below.

### 2.6. Photosynthetic Measurements

The yield of light-adapted Photosystem-II (PSII) electron transport (Φ_PSII_) was measured on intact plants in the fume-hoods using a portable chlorophyll fluorometer (model OS1-FL, Opti-Sciences, Hudson, NH, USA). For leaf pieces, light-adapted Φ_PSII_ and dark-adapted (>30 min) maximum PSII efficiency (F_v_/F_m_) was measured with a pulse-modulated bench-top chlorophyll fluorometer (model 101/103, Walz, Germany) and a flash lamp (model KL1500 LCD, Schott, Germany; 2-s flash at ca. 6000 μmol m^−2^ s^−1^ PAR). Leaf relative chlorophyll concentration [chl] was measured using a portable SPAD meter (model SPAD-502 Plus, Konica Minolta, Tokyo, Japan). Leaf gas-exchange measurements were made with a portable infra-red gas-analyzer (IRGA)-based system with a 2 × 3-cm cuvette with red/blue LED light (model LI-6400, LiCor, Lincoln, NE, USA). Net photosynthesis (P_n_ = net CO_2_ uptake), stomatal conductance to water vapor (G_s_), and internal CO_2_ concentration (C_i_) measurements were made on intact plants at 400 ppm CO_2_ (excluding P_n_–C_i_ curves discussed below), 1000 μmol m^−2^ s^−1^ PAR, and 25 or 28 °C air temperature (for lettuce and corn, respectively). For single measurements at 400 ppm CO_2_, data were collected within ca. 1 min of inserting leaves into the cuvette (immediately after readings stabilized). All photosynthetic measurements were made between 11 am and 3 pm on the middle of the most recently expanded leaf (corn) or on the outer half of a recently fully expanded leaf (lettuce); all measurements except for F_v_/F_m_ were made on leaves receiving steady-state illumination (the actinic light level for Φ_PSII_ measurements)

In vivo rubisco carboxylation activity was determined using the “A-C_i_” technique [49]. Briefly, P_n_ and C_i_ were measured repeatedly on individual intact lettuce plants at each of the following CO_2_ concentrations: 400, 250, 200, 150, and 100 ppm. The light level and the temperature were 1000 μmol m^−2^ s^−1^ PAR and 25 °C, respectively. Results for each plant were graphed individually, with P_n_ on the *Y*-axis and C_i_ on the *X*-axis. The initial linear slope of the P_n_–C_i_ relationship, a measure of in vivo rubisco activity in C3 plants, was determined for 100–250 ppm CO_2_ (r^2^ > 0.98 for all curves). Data were collected after readings stabilized at each CO_2_ level (within 2–3 min).

As an additional method to test for direct effects of cyanobacterial toxins on photosynthesis, the effects of toxins on in vitro O_2_ evolution of thylakoid membranes were investigated (experiment 8). Partially intact thylakoids were isolated from tomato (*Solanum lycopersicum* L. ‘Celebrity’) leaves using a simplified version of the method described in [50]. Tomato was used because of its high concentration of chlorophyll and the ease with which chloroplasts are isolated from its leaves. Briefly, leaf tissue was homogenized in a blender with chloroplast buffer (350 mM sorbitol, 50 mM HEPES (pH 7.75), 5 mM EDTA (ethylene-diamine-tetra-acetic acid), 5 mM ascorbate, 2 mM dithiothreitol, and 2% polyvinylpyrrolidone), filtered through a cotton cloth, and centrifuged for 10 min at 500× *g* to remove intact cells and nuclei. The supernatant was layered onto 1M sucrose in 50 mM HEPES (pH 7.75) and then centrifuged in a swinging-bucket rotor for 10 min at 5000× *g.* The resulting crude chloroplast pellet was resuspended in 100 mM sucrose (resulting in hypotonic lysis, producing a thylakoid preparation) and frozen at −80 °C until further use. Isolated thylakoids were resuspended to 25 µg chlorophyll per mL in thylakoid resuspension buffer (4 mM magnesium chloride, 4 mM sodium chloride, 50 mM HEPES, and 0.01% Triton, pH 7.75). Thylakoid preparations were dispensed into 50 mL plastic “Falcon” tubes (5 replicate tubes per treatment), to which was added one of four toxins (ANA, BMAA, LPS, or MC-LR) to a final concentration of 1 µM toxin (ANA, BMAA, MC-LR) or 50 µg mL^−1^ (LPS); deionized water was added to the control group. Potassium ferricyanide (K_3_Fe(CN)_6_) was added to 4 mM to each tube as an artificial electron acceptor. After 15 min under 130–140 µmol m^−2^ s^−1^ PAR, the dissolved O_2_ concentration was measured using a portable galvanic-type O_2_-electrode meter (model HI 9142, Hannah Instruments, Woonsocket, RI, USA). After 25 min, another dissolved O_2_ reading was taken, from which the rate of O_2_ evolution was calculated.

### 2.7. Statistical Analysis

All statistics were computed in R version 4.3.2 [51]. Comparisons of means were analyzed with *t*-test (for two comparisons) or ANOVA (for more than two comparisons); unless otherwise stated, *n* = 4–6, depending on the experiment. Homogeneity of variance and normality distributions were assessed with Levene and Shapiro–Wilk tests, respectively. If variance was unequal between groups, a Welch’s corrected t-test or ANOVA was performed instead (noted in the results). Fisher’s LSD post hoc tests were performed for ANOVAs with a *p*-value ≤ 0.05. For Welch’s corrected ANOVA, selected *t*-tests were used to evaluate biologically significant differences among means. Multivariate comparisons were visualized for lettuce grown hydroponically in toxins or cyanobacterial extracts using principal component analysis (PCA). Individual plant replicates were plotted on two orthogonal principal component axes. PSII yield (Φ_PSII_) was excluded from the response variables used for PCA because it limited replicate inclusion and there were not biologically or statistically significant differences in Φ_PSII_ among treatments. All other measured response variables were included and plotted as vectors indicating the direction and magnitude of their effects on multivariate differences among individuals. Treatment groups are indicated with hull polygons for clarity.

## 3. Results

### 3.1. Cyanobacterial Culture Effects on Plant Photosynthesis

**Figure 1:**
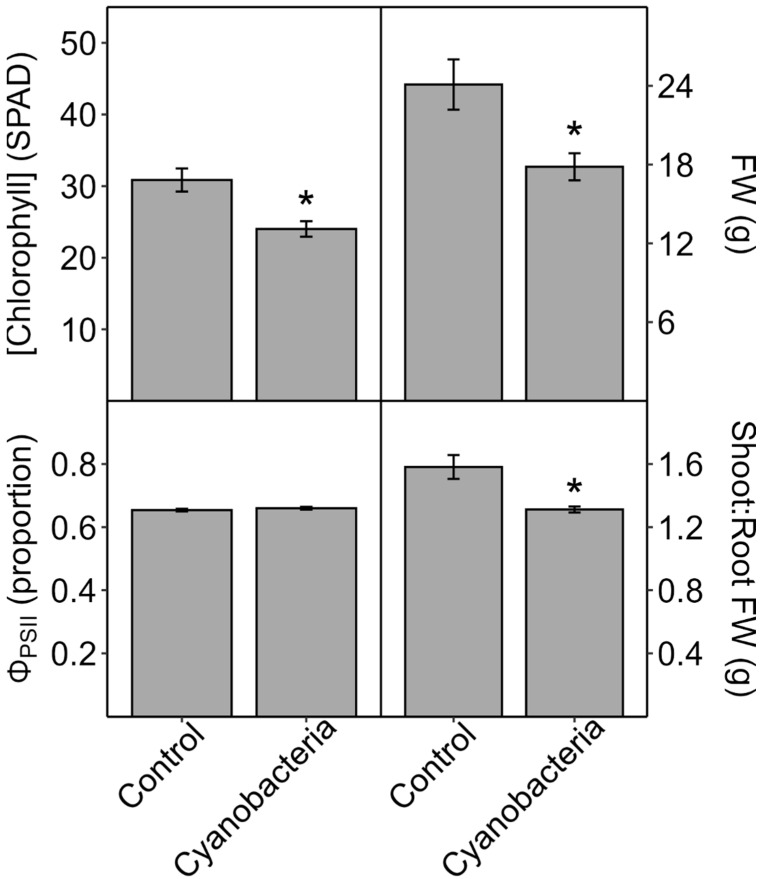
Effects of *Microcystis aeruginosa* culture (intact cells) on soil-grown corn (*Zea mays*) after 14 days. Roots of potted plants grown in the greenhouse were watered every third day with nutrient solution only (control) or nutrient solution containing live cyanobacteria. Just prior to harvest to obtain plant fresh weight (FW) and shoot-to-root FW ratio, yield of light-adapted Photosystem-II electron transport (Φ_PSII_) and relative chlorophyll concentration ([chlorophyll] (SPAD)) were measured on recently expanded leaves of intact plants. Results are means ± 1 SE, *n* = 5–6, and significant treatment differences at *p* ≤ 0.05 (*t*-test) are indicated with an asterisk.

**Figure 2:**
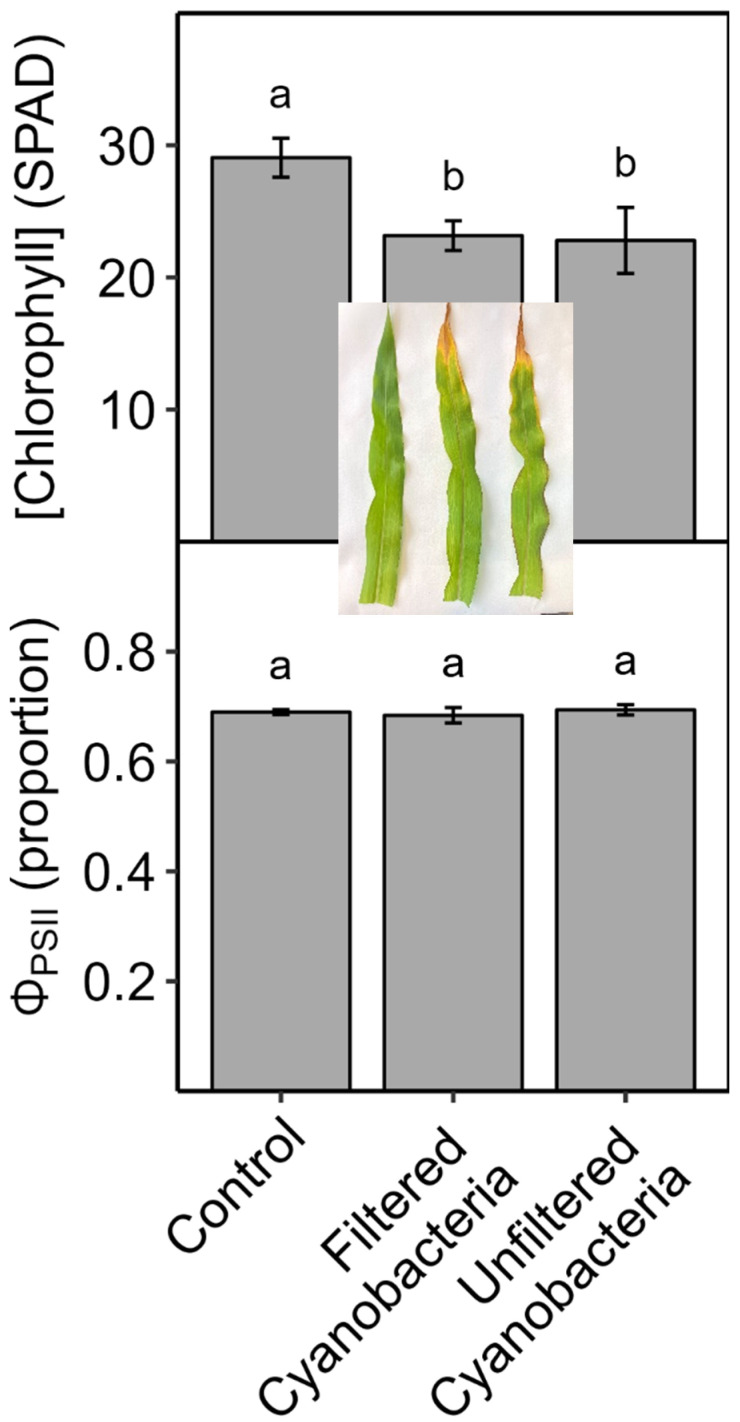
Effects of *Microcystis aeruginosa* culture (with or without intact cells) on soil-grown corn (*Zea mays*) plants after 14 days. Plants were grown and watered as in Figure 1 with nutrient solution only (control) or nutrient solution containing cell-free (filtered) or intact-cell (unfiltered) cyanobacterial culture. Yield of light-adapted Photosystem-II electron transport (Φ_PSII_) and relative chlorophyll concentration ([chlorophyll] (SPAD)) were measured on recently expanded leaves of intact plants. Results are means ± 1 SE, *n* = 5, and significant treatment differences at *p* ≤ 0.05 (ANOVA followed by LSD test) are indicated with different letters above bars. Insert photo shows leaves (ca. 3 cm wide) from each treatment after 14 d in the same order as the *X*-axis.

**Figure 3:**
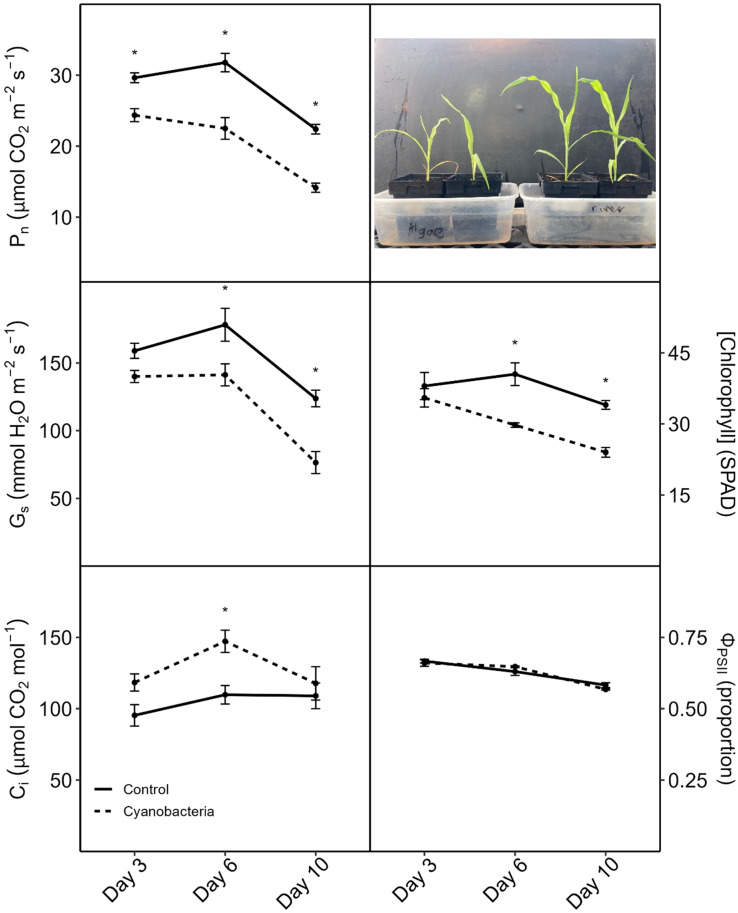
Effects of *Anabaena flos-aquae* culture (cells lysed and removed) on soil-grown corn (*Zea mays*) plants after 10 days. Plants were grown and watered as above with nutrient solution only (control) or nutrient solution containing cell-free (cells lysed and then filtered) cyanobacterial culture. Net photosynthesis (P_n_, CO_2_ uptake), stomatal conductance (G_s_), internal CO_2_ concentration (C_i_), yield of light-adapted Photosystem-II electron transport (Φ_PSII_), and relative chlorophyll concentration ([chlorophyll] (SPAD)) were measured on recently expanded leaves of intact plants. Results are means ± 1 SE, *n* = 4, and significant treatment differences between treatments within each day at *p* ≤ 0.05 (*t*-test) are indicated with an asterisk. Insert photo shows plants from each treatment, with the control on the right.

Cyanobacterial culture treatment of soil-grown corn for 10–14 d: When roots of corn plants grown in soil were watered every 3 days for 14 days with *M. aeruginosa* culture (whole cells in nutrient solution), the plant biomass, shoot-to-root mass ratio, and leaf [chl] were reduced compared to control plants watered with only nutrient solution, while Φ_PSII_ was not affected (Figure 1). Similar results were obtained in a follow-up experiment wherein for soil-grown corn plants that were watered with either whole-cell or cell-free *M. aeruginosa* culture, leaf [chl], but not Φ_PSII_, was reduced compared to control plants after 14 days (Figure 2). In a third experiment with soil-grown corn watered with *A. flos-aquae* culture (cells lysed and removed), P_n_ and G_s_ decreased and C_i_ increased within 3 days compared to the controls, while leaf [chl] did not decrease until day 6, and Φ_PSII_ did not change within 10 days (Figure 3). Decreases in P_n_, G_s_, and leaf [chl] became progressively larger over the course of the experiment, and they were accompanied by increases in C_i_ relative to the controls, indicating that decreases in P_n_ were not driven by decreases in G_s_. Biomass was also reduced by the cyanobacteria treatment (Figure 3, inset; total plant fresh mass (g), means ± 1 S.E.: controls = 0.44 ± 0.48, + cyanobacteria = 0.35 ± 0.010).

**Figure 4:**
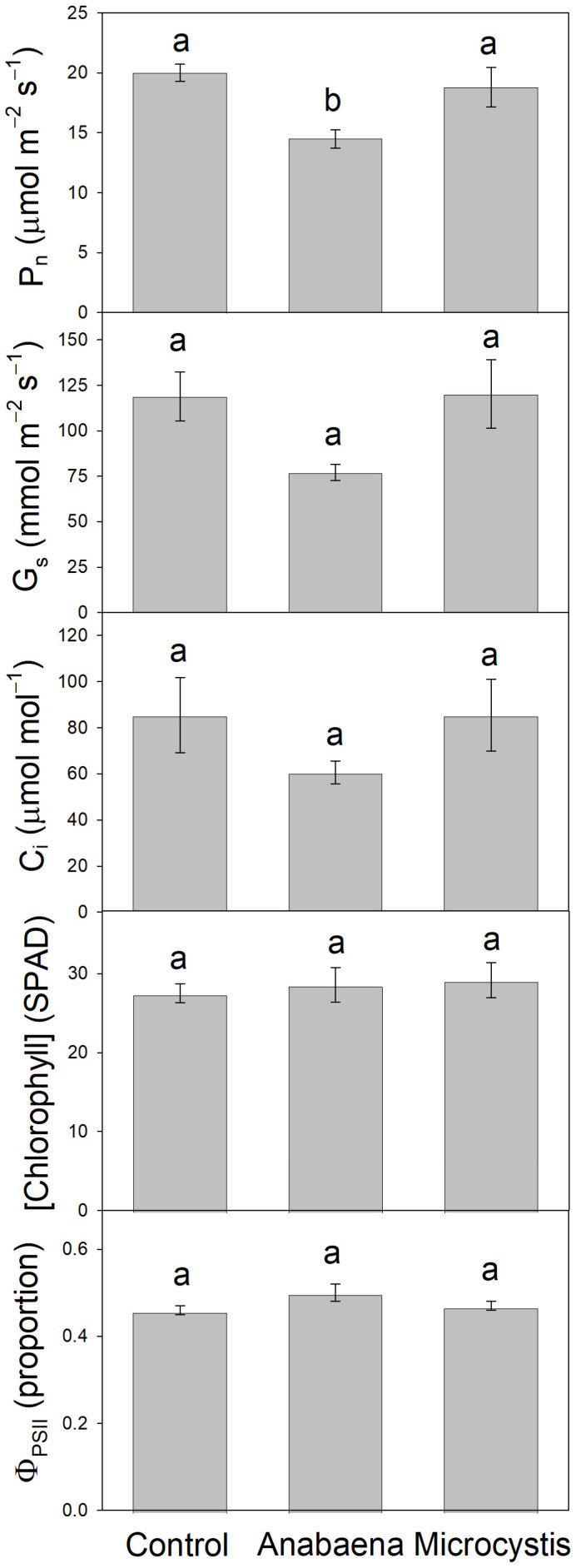
Effects of *Anabaena flos-aquae* or *Microcystis aeruginosa* culture (cells lysed and removed) on hydroponic corn (*Zea mays*) plants after 24 h. Plants were grown in soil and transferred to glass containers with roots submerged in nutrient solution only (control) or nutrient solution containing cell-free (cells lysed and then filtered) cyanobacterial culture. Net photosynthesis (P_n_, CO_2_ uptake), stomatal conductance (G_s_), internal CO_2_ concentration (C_i_), yield of light-adapted Photosystem-II electron transport (Φ_PSII_), and relative chlorophyll concentration ([chlorophyll] (SPAD)) were measured on recently expanded leaves of intact plants. Results are means ± 1 SE, *n* = 4–6, and significant treatment differences at *p* ≤ 0.05 (ANOVA followed by LSD test) are indicated with different letters above the bars.

Cyanobacterial culture treatment of hydroponic corn treated for 24 h: When roots of young corn plants were submerged for 24 h in either *M. aeruginosa* or *A. flos-aquae* culture (cells lysed and removed), P_n_ and G_s_ decreased in *A. flos-aquae*-treated plants, while C_i_ increased (non-significantly for G_s_ and C_i_); P_n_, G_s_, and C_i_ were not affected in *M. aeruginosa*-treated plants within this time frame (Figure 4). Neither Φ_PSII_ nor leaf [chl] were affected by either cyanobacterial culture within 24 h. 

**Figure 5:**
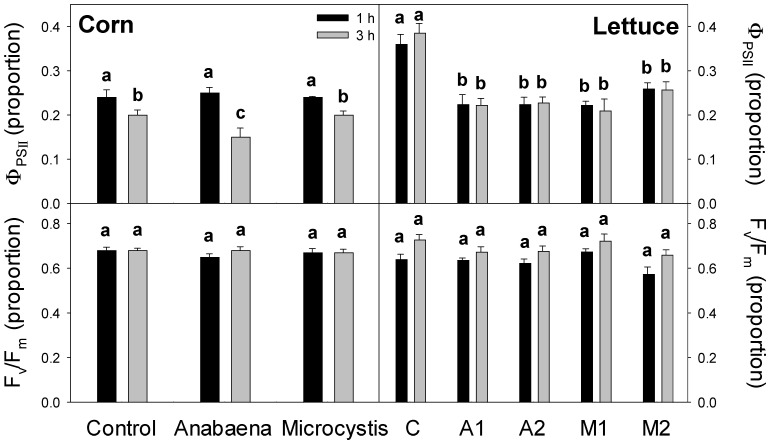
Effects of *Anabaena flos-aquae* or *Microcystis aeruginosa* culture (cells lysed and removed) on leaf pieces of corn (*Zea mays*) or lettuce (*Lactuca sativa*) after 1 or 3 h. Leaf pieces were vacuum-infiltrated with nutrient solution only (control, C) or nutrient solution containing cyanobacterial culture (*Anabaena*, A; *Microcystis*, M). For lettuce, two independent cultures of each species were tested (A1, A2; M1, M2). The yields of light-adapted Photosystem-II (PSII) electron transport (Φ_PSII_) and dark-adapted maximum PSII efficiency (F_v_/F_m_) were measured on leaf tissue from recently expanded leaves (for Φ_PSII_, 750 ± 25 µmol m^−2^ s^−1^ PAR). Results are means + 1 SE, *n* = 5, and significant treatment differences at *p* ≤ 0.05 (ANOVA followed by LSD test) are indicated with different letters above bars.

Cyanobacterial culture treatment of infiltrated corn and lettuce leaves for 1–3 h: When leaf discs of corn and lettuce were vacuum-infiltrated with either *M. aeruginosa* or *A. flos-aquae* culture (cells lysed and removed), Φ_PSII_ was reduced compared to controls within 3 h in corn treated with *A. flos-aquae* but not with *M. aeruginosa* (Figure 5). In lettuce, Φ_PSII_ was reduced compared to controls within 1 h in both *M. aeruginosa* and *A. flos-aquae* treated plants.

### 3.2. Individual Toxin Effects on Plant Photosynthesis

**Figure 6:**
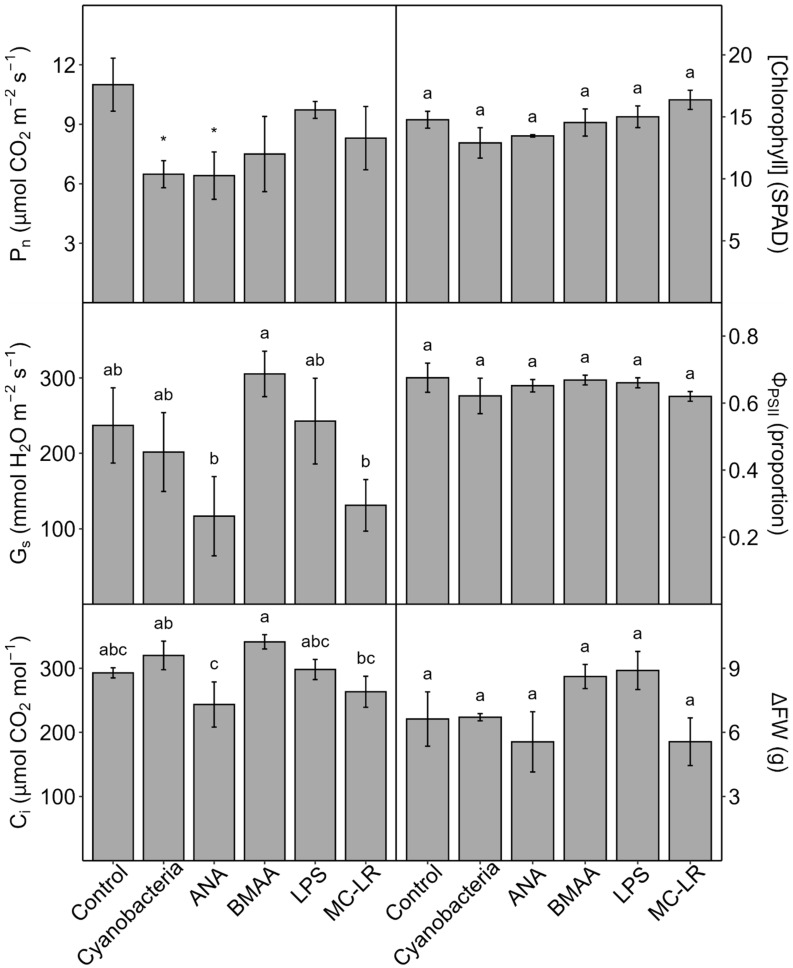
Effects of *Anabaena flos-aquae* culture or pure cyanobacterial toxins on hydroponic lettuce (*Lactuca sativa*) plants after 10 days. Plants were grown in soil and transferred to glass containers with roots submerged in nutrient solution only (control), cyanobacterial culture (cells lysed and removed from nutrient solution), or individual toxins in nutrient solution (0.5 μM: anatoxin-a, ANA, beta-methyl-amino-alanine, BMAA, microcystin-LR; 25 µg mL^−1^: lipopolysaccharide, LPS). Just prior to harvest to obtain the increase in plant fresh weight during treatment (ΔFW), net photosynthesis (P_n_, CO_2_ uptake), stomatal conductance (G_s_), internal CO_2_ concentration (C_i_), yield of light-adapted Photosystem-II electron transport (Φ_PSII_), and relative chlorophyll concentration ([chlorophyll] (SPAD)) were measured on recently expanded leaves of intact plants. Results are means ± 1 SE, *n* = 4. Significant treatment differences at *p* ≤ 0.05 (ANOVA followed by LSD test) are indicated with different letters above bars, except for P_n_, where significant differences (Welch’s ANOVA and *t*-test) from the control are indicated with asterisks (see Section 2).

**Figure 7:**
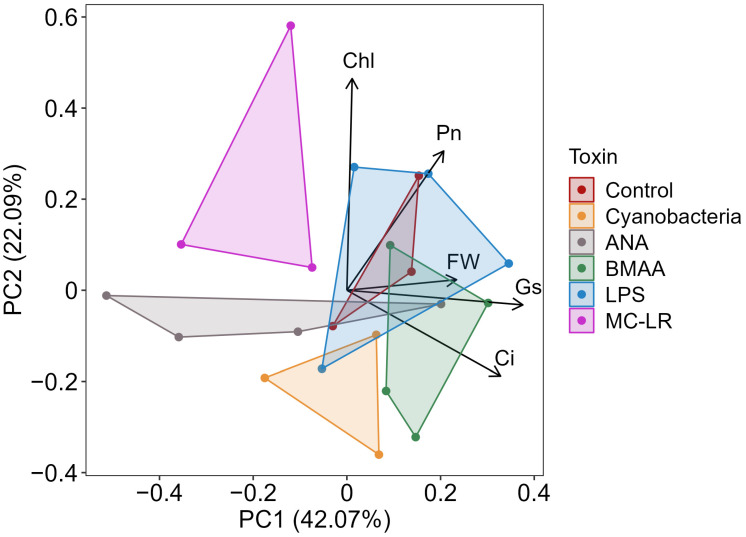
Principal components analysis (PCA) visualizing overall differences in the responses of lettuce (*Lactuca sativa*) to *Anabaena flos-aquae* culture and individual toxins from Figure 6. Vectors represent relative chlorophyll concentration (Chl), net photosynthesis (P_n_), increase in fresh weight (FW), stomatal conductance (G_s_), and internal CO_2_ concentration (C_i_). Points represent eigen-values of individual replicate plants. Differences in replicates are visualized based on their position on two orthogonal principal component axes. The direction and magnitude of response-variable vectors indicate their direction and degree of effect on the replicate position. Treatments are indicated by color: nutrient solution only (control); cyanobacterial culture, cells lysed and removed from nutrient solution (Cyanobacteria); or individual toxins in nutrient solution (0.5 μM: anatoxin-a, ANA, beta-methyl-amino-alanine, BMAA, microcystin-LR, MC-LR; 25 µg mL^−1^: lipopolysaccharide, LPS).

Individual toxin (and cyanobacterial-culture) treatments of hydroponic lettuce for 10 d: When roots of young lettuce plants were submerged for 10 days in either *A. flos-aquae* culture (cells lysed and removed) or individual pure toxins (ANA, BMAA, LPS, or MC-LR), P_n_ was reduced compared to the controls (the decreases were significant only with cyanobacterial culture and anatoxin, wherein P_n_ decreased by 41%) (Figure 6). Large but non-significant decreases in G_s_ were observed with anatoxin and MC-LR, and G_s_ increased non-significantly with BMAA. Small non-significant changes in C_i_ were observed for anatoxin (decrease) and BMAA (increase); otherwise, there were no meaningful differences between treatments and controls. None of the treatments had significant effects on Φ_PSII_, leaf [chl], or growth (increase in fresh mass, ΔFM), though non-significant decreases in leaf [chl] were observed with cyanobacterial culture and anatoxin, and non-significant increases in growth were observed with BMAA and LPS. Because the number of replicates per treatment (and hence the power) was constrained in this experiment by logistics, we then included all of the response parameters in a clustering analysis (PCA) and found that all treatments except LPS caused divergence from the controls, especially cyanobacterial culture, anatoxin, and MC-LR, while the two-dimensional space for LPS expanded compared to the controls (Figure 7).

**Figure 8:**
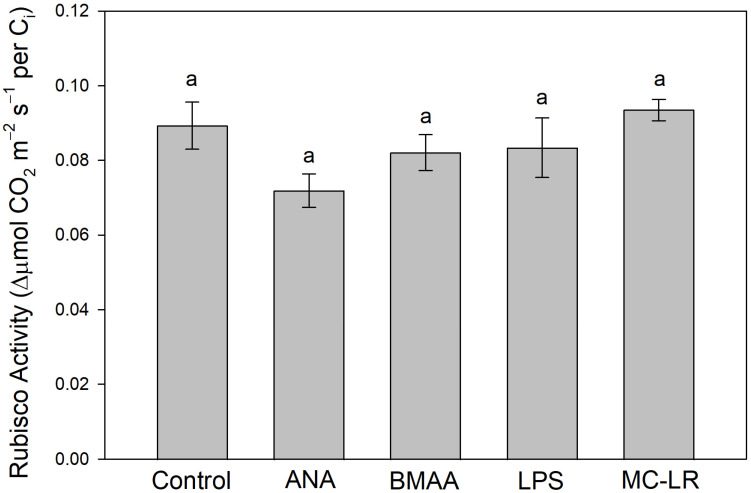
Effects of pure cyanobacterial toxins on in vivo rubisco activity in hydroponic lettuce (*Lactuca sativa*) plants after 10 days. Plants were grown and treated as in Figure 6, with roots submerged in nutrient solution only (control) or individual toxins in nutrient solution (0.5 μM: anatoxin-a, ANA, beta-methyl-amino-alanine, BMAA, microcystin-LR, MC-LR; 25 µg mL^−1^: lipopolysaccharide, LPS). Rubisco activity was determined from the initial slope of the photosynthesis–CO_2_ response curve, measured in recently expanded attached leaves at 1000 μmol m^−2^ s^−1^ PAR and 25 °C. Results are means ± 1 SE, *n* = 4. There were no significant treatment differences with ANOVA (*p* = 0.12), as indicated by the same letters above the bars, but ANA differed from the control with one-tailed *t*-test (*p* = 0.03).

The above experiment was then independently replicated (minus the cyanobacterial-extract treatment) to investigate the effects of individual toxins on in vivo rubisco activity, an indication of toxin effects on CO_2_-fixation reactions, and to confirm the general effects of toxins on P_n_, G_s_, and C_i_. In this second experiment, ANA reduced rubisco activity by 19.5%, while BMAA and LPS caused smaller (non-significant) reductions, and MC-LR caused no reduction (Figure 8). As in the first experiment, in the repeat experiment, P_n_ measured at 400 μmol/mol CO_2_ was reduced the most by ANA (35%), with smaller (non-significant) reductions for BMAA, LPS, and MC-LR; no significant effects of toxins on G_s_ and C_i_ were observed (Supplemental Table S1). To confirm the above effects of ANA on rubisco activity, we then measured the effects of ANA vs. the control on rubisco activity in another (third) independent experiment and found that ANA reduced rubisco activity by 17%.

**Figure 9:**
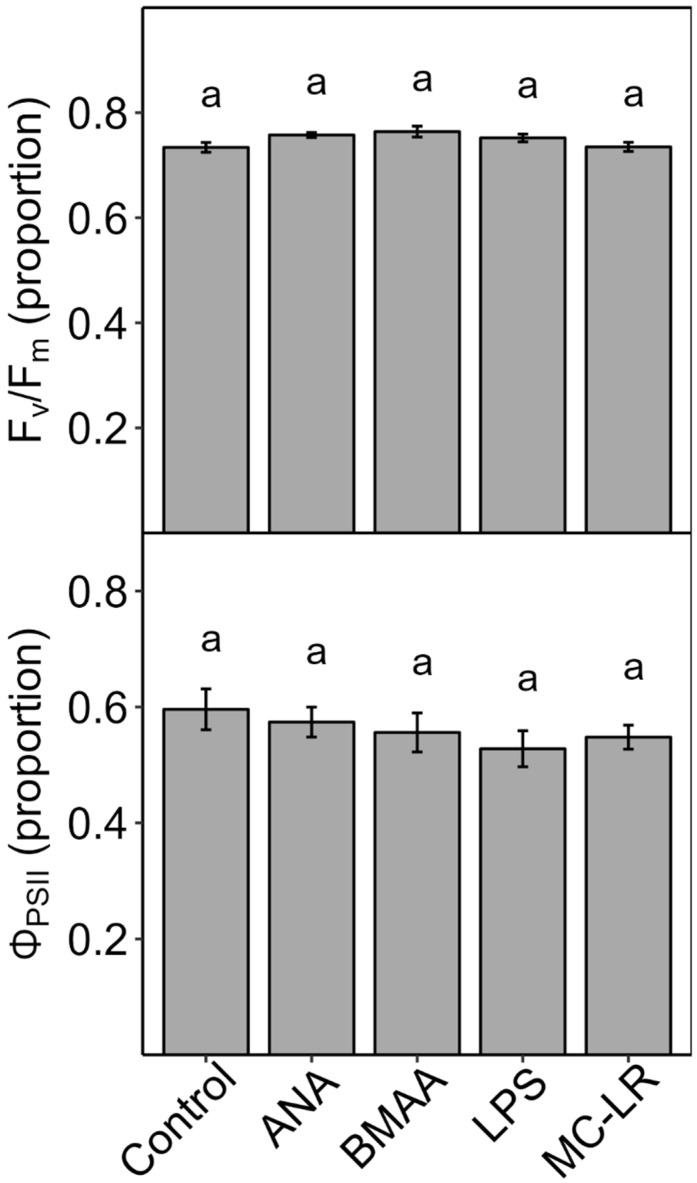
Effects of individual cyanobacterial toxins on detached leaves of lettuce (*Lactuca sativa*) after 2 h. Leaf pieces were vacuum-infiltrated with deionized water only (control) or individual toxins in deionized water (1 μM: anatoxin-a, ANA, beta-methyl-amino-alanine, BMAA, microcystin-LR, MC-LR; 50 µg mL^−1^: lipopolysaccharide, LPS). Leaves were then incubated for 2 h under 450 ± 25 µmol m^−2^ s^−1^ PAR or in the dark, after which the yield of light-adapted Photosystem-II (PSII) electron transport (Φ_PSII_) or dark-adapted maximum PSII efficiency (F_v_/F_m_) was measured. Results are means ± 1 SE, *n* = 5. There were no significant effects of toxins on Φ_PSII_ or F_v_/F_m_ (ANOVA, *p* > 0.05), as indicated by the same letters above the bars.

**Figure 10:**
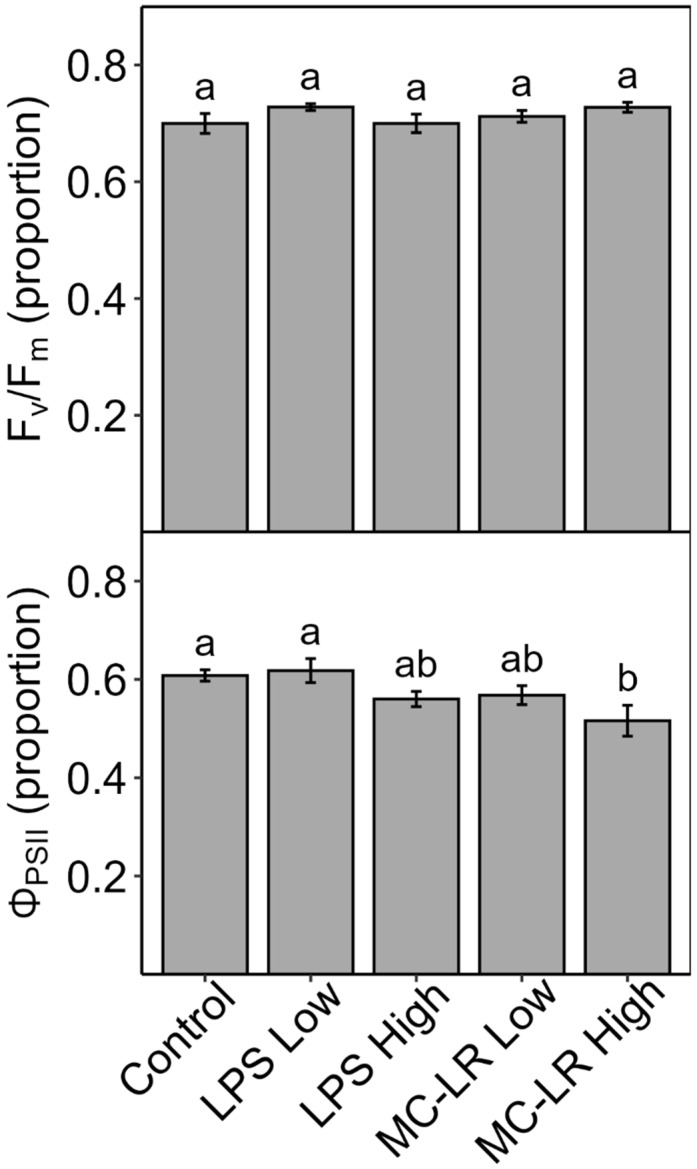
Effects of low vs. high concentrations of the cyanobacterial toxins, lipopolysaccharide (LPS), and microcystin-LR (MC-LR) on detached leaves of lettuce (*Lactuca sativa*) after 3 hours. Leaf pieces were vacuum-infiltrated with deionized water only (control) or 1 µM MC-LR (MC-LR Low), 10 µM MC-LR (MC-LR High), 50 µg mL^−1^ LPS (LPS Low), or 250 µg mL^−1^ LPS (LPS High). Leaves were then incubated for 3 h under 750 ± 25 µmol m^−2^ s^−1^ PAR or in the dark, after which the yield of light-adapted Photosystem-II (PSII) electron transport (Φ_PSII_) or dark-adapted maximum PSII efficiency (F_v_/F_m_) was measured. Results are means ± 1 SE, *n* = 5, and significant treatment differences at *p* ≤ 0.05 (ANOVA followed by LSD test) are indicated with different letters above bars.

Individual toxin treatment of infiltrated lettuce leaves infiltrated for 2–3 h: When leaf discs of lettuce were vacuum-infiltrated with individual pure toxins, neither dark-adapted F_v_/F_m_ (maximum PSII efficiency) nor light-adapted Φ_PSII_ (PSII yield) was significantly affected after 2 h, although the values for LPS and MC-LR were lowest among all treatments (Figure 9). In a follow-up experiment, when leaves were vacuum-infiltrated with LPS or MC-LR at a higher concentration, Φ_PSII_ decreased after 3 h (significant for MC-LR), but F_v_/F_m_ did not (Figure 10).

**Figure 11:**
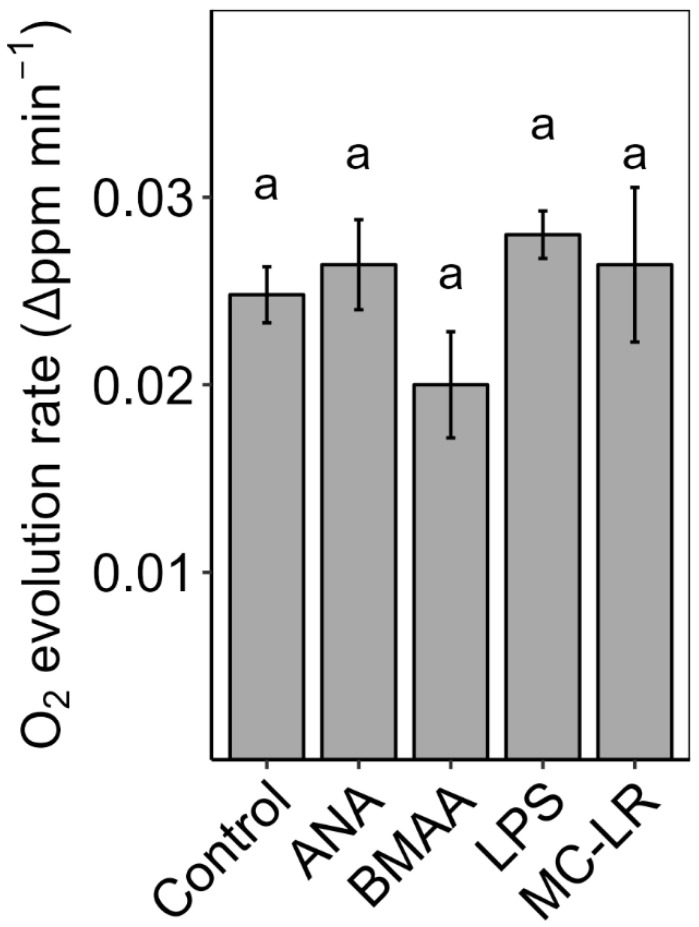
The effects of cyanobacterial toxins on in vitro O_2_ evolution of thylakoid membranes. Chloroplasts were isolated from tomato (*Solanum lycopersicum*) leaves and lysed to obtain thylakoids. Thylakoids were treated with either deionized water or individual toxins (1 µM: anatoxin-a, ANA, beta-methyl-amino-alanine, BMAA, microcystin-LR, MC-LR; 50 µg mL^−1^: lipopolysaccharide, LPS). Results are means ± 1 SE, *n* = 5. There were no significant effects of toxins on O_2_ evolution (ANOVA, *p* > 0.05), as indicated by the same letters above the bars.

Individual toxin treatment of tomato chloroplast membranes for ca. 1 h: When chloroplast membranes were treated with individual pure toxins, none of the toxins significantly affected photosynthetic O_2_ evolution, although the BMAA was lower than all other treatments (Figure 11).

## 4. Discussion

A limited number of previous studies have reported variable effects (negative to neutral to positive) of cHAB cell-extracts or toxins on one or more aspects of photosynthesis in cyanobacteria, eukaryotic algae, and aquatic and terrestrial plants (e.g., [4,9,16]). However, because these studies often differed in study organisms, toxins, dosage, duration, mode of exposure, and the photosynthetic response variable measured, it is not clear which cHAB toxins affect photosynthesis and how. To clarify if and how cHAB toxins affect photosynthesis in terrestrial plants, we investigated short-term and longer-term effects of cHAB extracts from two different cHAB species and four individual toxins on multiple aspects of photosynthesis in two different plant species exposed to toxins through roots or leaves.

When corn plants were grown in soil and watered with cHAB culture or extract, plant growth (Figure 1 and Figure 3), leaf chlorophyll concentration (Figure 1, Figure 2 and Figure 3), and net (overall) photosynthesis (P_n_, Figure 3) were reduced significantly, but the PSII yield (Φ_PSII_) was not affected (Figure 1, Figure 2 and Figure 3). The effects were observed with either *M. aeruginosa* or *A. flos-aquae* cyanobacteria and with whole-cell culture in nutrient solution, culture with cells removed, or culture with cells lysed and removed (the latter is functionally equivalent to cyanobacterial extract used in other studies). These cHAB-caused decreases in P_n_ were accompanied by decreases in stomatal conductance (G_s_, an index of stomatal opening), but the increases in leaf internal CO_2_ concentration (C_i_) indicate that declines in P_n_ were caused by damage to photosynthetic metabolism rather than stomatal closure. Because Φ_PSII_ is determined by PSII efficiency and electron transport downstream from PSII [52], the lack of toxin effects on Φ_PSII_ indicates that photosynthetic electron transport was not impacted in these experiments. Hence, the damage to photosynthesis was likely due to CO_2_-fixation (i.e., the Calvin Cycle) and/or due to the loss of chlorophyll. Because Φ_PSII_ reflects the relative performance of existing PSII centers independent of their concentration in the leaf, and this did not decrease, then the loss of chlorophyll per unit leaf area indicates a reduction in the concentration of PSII per unit area, which would contribute to a decrease in P_n_ (confirmed in Figure 3). However, decreases in P_n_ occurred prior to decreases in leaf [chl], indicating that some of the decrease in P_n_ was attributable to damage to the Calvin Cycle (e.g., rubisco activity, Figure 8). Similarly to this study, a decrease in P_n_ (measured as O_2_ evolution after 42 d of exposure) was observed in soil-grown spinach (*Spinacia oleracea*) watered with cHAB extract derived from *M. aeruginosa* and *Aphanizomenon flos-aquae* containing 0.5 μg/L MC-LR [45]. In contrast, cHAB extract derived from *M. aeruginosa* containing up to 22,000 μg/L MC-LR decreased the maximum PSII efficiency (dark F_v_/F_m_; measured after 30 d) in sand-grown tomato [46], while an *M. aeruginosa* extract containing up to 10 μg/L MC-LR increased P_n_ (measured after 15 d) in lettuce grown in bark and vermiculite [47].

Importantly, our experiments with soil-grown plants could not determine if the effects of cHAB cultures or extracts on photosynthesis were direct primary or indirect secondary effects, as decreases in leaf [chl] or P_n_ were not observed until after three or more days of treatment. To investigate if cHAB extracts had short-term, and thus possibly direct, effects on photosynthesis, we examined the effects on photosynthesis in (1) hydroponic corn plants with roots immersed for only 24 h in cHAB extract, and (2) corn and lettuce leaf tissue vacuum-infiltrated with cHAB extract for only 1 or 3 h. In the hydroponic corn, P_n_, G_s_, and C_i_ all decreased within 24 h, and leaf [chl] and Φ_PSII_ were unaffected (Figure 4). In the vacuum-infiltrated leaf tissue, light-adapted Φ_PSII_ decreased, but dark-adapted F_v_/F_m_ did not (Figure 5), indicating that damage to PSII was from photo-inhibition (light-dependent photo-oxidation). Interestingly, in these experiments, lettuce was sensitive to extracts from both *M. aeruginosa* and *A. flos-aquae*, while corn was sensitive to only *A. flos-aquae* extract. Rapid (≤1 h) effects of cHAB extracts on photosynthesis (i.e., Φ_PSII_) in some eukaryotic algae have also been observed [18,41]. Our results above indicate that some cHAB toxins can have rapid negative effects on photosynthesis in plants, but that the effects can differ depending on the mode of exposure (roots vs. leaves), which has relevance for the method used when irrigating crops with cHAB-contaminated water (e.g., drip vs. spray, as in [11]).

Because cHAB irrigation water, lab cultures, or cell extracts likely contain many toxins, including rarely measured toxic peptides besides MCs [19], any cHAB effects on photosynthesis cannot be attributed to any single toxin without additional investigation. Consequently, we then conducted several experiments wherein we treated plants with individual pure cHAB toxins. In hydroponic lettuce plants with roots exposed to cHAB extract or individual toxins for 10 d, P_n_ decreased (although not always significantly) in all treatments, especially cHAB (*A. flos-aquae*) extract and pure anatoxin-a (“Cyanobacteria” and “ANA” treatments in Figure 6 and Table S1). These decreases were not associated with damage to photosynthetic electron transport, except possibly in Cyanobacteria and MC-LR treatments (which had small non-significant decreases in Φ_PSII_) or due to loss of chlorophyll, except possibly in Cyanobacteria and ANA treatments (which had small non-significant decreases). The decreases in P_n_ were also not associated with stomatal closure, with the possible exception of ANA (Figure 6), BMAA, (Table S1), and MC-LR (Figure 6 and Table S1), as indicated by small non-significant decreases in C_i_. Damage to the CO_2_-fixation reactions of photosynthesis was suggested by non-significant increases in C_i_ with Cyanobacteria and BMAA treatments (Figure 6), and by decreases in rubisco activity with ANA (and, to a lesser extent, BMAA and LPS) (Figure 8). Results from the two replicate experiments with hydroponic lettuce indicate that (1) cHAB extract and all four cHAB toxins negatively affected photosynthesis in some way; (2) neither cHAB extract nor any individual toxin had a single main lesion to photosynthesis; and (3) all five treatments (cHAB extract and four toxins) each affected photosynthesis in different ways. The latter conclusion was confirmed with a multi-variate statistical analysis (PCA), which showed that Cyanobacteria (extract), ANA, BMAA, MC-LR, and the control sorted almost completely independently of each other, and LPS overlapped with the control but had an expanded two-dimensional space (Figure 7). Our results are consistent with past studies, most of which observed negative effects of individual cHAB toxins on photosynthesis, e.g., MC-RR in cyanobacteria (decrease in chl a, phycocyanin; [40]); MC-LR, -RR, -YR, and -LF in various eukaryotic algae (decrease in Φ_PSII_; [18,41]); MC-LR in soybean (*Glycine max*) (decrease in P_n_; [44]) and cat-tail (*Typha angustifolia*) (decreases in P_n_, G_s_, C_i_, chl, in vitro rubisco activity; [43]); ANA in *Lemna* species (decrease in P_n_, chl; [25,27]) and *Ceratophyllum demersum* (decrease in chl; [26]); and LPS in *Arabidopsis thaliana* (decrease in F_v_/F_m_; [36]).

As with our experiments with cHAB extract, we conducted experiments with individual cHAB toxins in which we vacuum-infiltrated leaf tissue to determine if any of the toxins had short-term effects on photosynthesis (i.e., Φ_PSII_). At relatively low concentrations of toxin (50 μg mL^−1^ LPS, 1 μM ANA/BMAA/MC-LR) and light (450 ± 25 µmol m^−2^ s^−1^ PAR), neither light-adapted Φ_PSII_ nor dark-adapted F_v_/F_m_ were significantly affected, although Φ_PSII_ was lowest in LPS and MC-LR (Figure 9). To follow up, we then examined the effects of LPS and MC-LR at higher concentrations (250 μg mL^−1^ LPS, 10 μM MC-LR) and with brighter light (750 ± 25 µmol m^−2^ s^−1^ PAR), which resulted in larger decreases in Φ_PSII_ but (still) no effects on F_v_/F_m_ (Figure 10). Hence, as with cHAB extract, the negative effects of LPS and MC-LR on PSII were associated with photo-inhibitory damage to PSII, and this damage increased with the dosage. Similarly to this study, Perron et al. [18] found that MC caused little decrease in maximum PSII efficiency (equivalent to our F_v_/F_m_) in all four species of eukaryotic algae examined but large decreases in PSII yield (equivalent to our Φ_PSII_) and that damage to Φ_PSII_ was dose-dependent. Also, as noted above, Shang-Guan et al. [36] found that LPS decreased PSII function, although, in their case, F_v_/F_m_.

Because neither cHAB extracts nor individual toxins affected the maximum PSII efficiency (dark-adapted F_v_/F_m_) and only affected the PSII yield (light-adapted Φ_PSII_), there was no indication of a direct immediate effect of cHAB toxins on photosynthesis in our experiments. To confirm this, we exposed isolated chloroplast thylakoids to individual toxins (ANA/BMAA/LPS/MC-LR), and none of the toxins decreased in vitro photosynthetic O_2_ evolution significantly (Figure 11). Therefore, our experiments indicated that the negative effects of cHAB toxins were not the consequence of direct immediate effects on photosynthetic metabolism but were rather due to indirect secondary effects, such as decreases in plant N status leading to decreases in the concentration of chlorophyll and other photosynthetic machinery, oxidative stress, such as photo-inhibition, and/or stomatal closure limiting CO_2_ uptake. As with cHAB extracts noted above, some past studies have found that individual cHAB toxins can have rapid (≤1 h) effects on photosynthesis. For example, both Perron et al. [18] and Garcia-Espin et al. [41] observed rapid effects of MC on Φ_PSII_ in eukaryotic algae, and Abe et al. [27] and Kaminski et al. [44] observed such effects on P_n_ in soybean with MC and *Lemna triscula* with ANA, respectively. Hence, direct effects of cHAB toxins on photosynthetic machinery cannot be ruled out yet.

As we expected, in our experiments, there were indications that whole-cell cHAB culture or extract had more severe effects on photosynthesis than individual toxins. First, decreases in Φ_PSII_ were greater for cHAB extract than for any individual toxin (Figure 5 vs. Figure 9 and Figure 10). Second, decreases in P_n_ were greatest for cHAB extract compared to all individual toxins except for ANA (which caused a similar decrease) (Figure 6). In contrast, Perron et al. [18] found similar decreases in the green alga, *Chlorella vulgaris*, in Φ_PSII_ for pure MC-LR and an amount of *M. aeruginosa* extract containing an equal concentration of MC-LR (and no significant levels of other MCs). Garcia-Espin et al. [41] also found that decreases in Φ_PSII_ were greater for two different cHAB extracts than for pure MC-LR in the four eukaryotic algae species examined.

In general, the two species examined in our study, corn and lettuce, showed similar photosynthetic responses to cHAB toxins, with some exceptions. In hydroponic plants of both species, cHAB extract from *A. flos-aquae* decreased P_n_ significantly, with smaller or no effects on Φ_PSII_ (Figure 4 vs. Figure 6), and it decreased Φ_PSII_ significantly in vacuum-infiltrated leaf tissue (Figure 5). However, as noted above, Φ_PSII_ in lettuce was more sensitive than in corn to cHAB extract from *M. aeruginosa* (Figure 5). It should also be noted that growth in soil-grown corn was very sensitive to cHAB extract (Figure 1 and Figure 3) but was unaffected in hydroponic lettuce, despite similar decreases in P_n_ (Figure 3 vs. Figure 6). Both Perron et al. [18] and Garcia-Espin et al. [41] found that sensitivity of Φ_PSII_ to cHAB toxins varied among species of eukaryotic algae, and Pflugmacher et al. [45] found variation among spinach varieties as far as sensitivity of P_n_ to cHAB extract.

## 5. Conclusions

Research to date has yielded conflicting results regarding the effects of cyanobacterial toxins on photosynthesis. We investigated short- to longer-term effects of cyanobacterial cultures/extracts and four individual toxins (anatoxin-a, beta-methyl-L-amino alanine, lipopolysaccharides, microcystin-LR) on multiple aspects of photosynthesis in two species of terrestrial plants (corn/*Zea mays*, lettuce/*Lactuca sativa*) exposed to toxins through roots or leaves. We found that at ecologically relevant concentrations, (1) cyanobacterial culture/extract and all four toxins negatively affected photosynthesis, although in different ways; (2) neither cyanobacterial culture/extract nor any individual toxin had a single main lesion to photosynthesis; (3) the mode of toxin exposure mattered, as the exposure of roots to toxins decreased photosynthetic CO_2_-fixation (i.e., the Calvin Cycle) and/or chlorophyll concentration but not photosynthetic electron transport (P_et_), while direct treatment of leaves reduced P_et_ in a light-dependent manner (photo-inhibition); and (4) in general, photosynthesis in corn and lettuce responded similarly to cyanobacterial cultures and toxins. These results improve our understanding of the negative effects of cyano-toxins on plants, which has implications for both growers and consumers of crops irrigated with cHAB water.

## Figures and Tables

**Table 1:** Summary of experiments and their key details.

Experiment	Treatment	Species	System	Explanation	Duration	Variables ^2^
1	cyanobacterial culture	corn	whole plants in soil	roots watered with *M. aeruginosa* whole-cell culture	14 d	Φ_PSII_, [chl], biomass
2	cyanobacterial culture	corn	whole plants in soil	roots watered with *M. aeruginosa* whole-cell vs. cell-free culture	14 d	Φ_PSII_, [chl]
3	cyanobacterial culture	corn	whole plants in soil	roots watered with *A. flos-aquae* lysed cell-free culture	10 d	P_n_, G_s_, C_i_, Φ_PSII_, [chl], biomass
4	cyanobacterial culture	corn	whole plants in hydroponics	roots submerged in *A. flos-aquae* or *M. aeruginosa* lysed cell-free culture	24 h	P_n_, G_s_, C_i_, Φ_PSII_, [chl]
5	cyanobacterial culture	corn, lettuce	leaf tissue	vacuum-infiltrated with *A. flos-aquae* or *M. aeruginosa* lysed cell-free culture	1–3 h	Φ_PSII_, F_v_/F_m_
6a and b	cyanobacterial culture and purified toxins	lettuce	whole plants in hydroponics	roots submerged in *A. flos-aquae* lysed cell-free culture (6a only) or individual toxins (ANA, BMAA, LPS, MC-LR) ^1^	10 d	P_n_, G_s_, C_i_, Φ_PSII_, [chl], biomass, (6b only) rubisco activity
7a and b	purified toxins	lettuce	leaf tissue	vacuum-infiltrated with individual toxins (ANA, BMAA, LPS, MC-LR) ^1^	1–3 h	Φ_PSII_, F_v_/F_m_
8	purified toxins	tomato	thylakoid membranes	individual toxins (ANA, BMAA, LPS, MC-LR) ^1^	1 h	in vitro O_2_ evolution

^1^ anatoxin-a = ANA; beta-methyl-amino-alanine = BMAA; lipopolysaccharide = LPS; microcystin-LR = MC-LR. ^2^ Φ_PSII_ = light-adapted Photosystem-II yield; [chl] = relative leaf chlorophyll concentration; F_v_/F_m_ = dark-adapted maximum Photosystem-II efficiency; P_n_ = net photosynthesis (CO_2_ uptake); G_s_ = stomatal conductance; C_i_ = leaf internal CO_2_ concentration.

## Data Availability

Datasets are available upon request.

## Supplementary Materials

The supporting information (Table S1) can be downloaded at: https://www.mdpi.com/article/10.3390/plants13223190/s1. Table S1: Results from Experiment 6b-Effects of pure toxins on leaf-level photosynthesis (Pn; μmol CO_2_ m^−2^ s^−1^), stomatal conductance (Gs; mmol m^−2^ s^−1^), and internal [CO_2_] (mol/mol) in hydroponic lettuce (Lactuca sativa) plants after 10 days.
