# Supplementary material for: Cyanobacterial Cultures, Cell Extracts, and Individual Toxins Decrease Photosynthesis in the Terrestrial Plants Lactuca sativa and Zea mays
Source: Plants (Basel). 2024 Nov 13;13(22):3190. doi: 10.3390/plants13223190 (PMC11597909; doi:10.3390/plants13223190)
Supplement: Supplementary file 1 [file plants-13-03190-s001.zip › plants-3174454-supplementary.pdf]

Supplemental Materials

Table S1- Results from Experiment 6b[1]- Effects of pure toxins[2] on leaf-level photosynthesis[3] (P_n; μmol CO_2 m^{-2} s^{-1}), stomatal conductance (G_s; mmol m^{-2} s^{-1}), and internal [CO_2] (μmol/mol) in hydroponic lettuce (*Lactuca sativa*) plants after 10 days.

	Control	ANA	BMAA	LPS	MC-LR
P_n	10.6+0.98^{a*}	6.9+0.03^{a*}	8.2+1.26^{a}	7.5+1.39^{a}	8.1+1.09^{a}
G_s	184+17.6^{a}	104+10.2^{a}	123+29.7^{a}	142+57.6^{a}	112+21.0^{a}
C_i	277+8.9^{a}	268+9.9^{a}	258+11.0^{a}	273+13.4^{a}	256+4.8^{a}

[1]Experiment 6b was an independent replicate of 6a, but without a cyanobacterial-extract treatment. Data are means + 1 SE, n = 4. Significant treatment differences ($P \leq 0.05$, ANOVA followed by LSD test) are indicated with different letters; for P_n, significant differences ($P = 0.029$, Mann-Whitney test) between control and ANA are indicated with asterisks.

[2]Treatments: Control = roots in nutrient solution only; roots in nutrient solution with: ANA = anatoxin-a (0.5 μM); BMAA = beta-methyl-amino-alanine (0.5 μM); MC-LR = microcystin-LR (0.5 μM); LPS = lipopolysaccharide (25 μg mL^{-1}).

[3]Measured at 400 μmol CO_2, 1000 μmol m^{-2} s^{-1} PAR, and 25°C.
